# Time-weighted lactate and glucose–lactate ratio outperform static values in ICU mortality prediction after traumatic brain injury: a retrospective cohort study

**DOI:** 10.1186/s40560-026-00864-9

**Published:** 2026-02-07

**Authors:** Matthias Manfred Deininger, Magdalena Ralser, Nico Haehn, Marius Huehn, Dmitrij Ziles, Gernot Marx, Catharina Conzen-Dilger, Anke Hoellig, Thomas Breuer

**Affiliations:** 1 Department of Intensive Care Medicine, Faculty of Medicine, RWTH Aachen University, Pauwelsstr. 30, 52074 Aachen, Germany; 2 Department of Neurosurgery, Faculty of Medicine, RWTH Aachen University, Pauwelsstr. 30, 52074 Aachen, Germany

**Keywords:** Traumatic brain injury, Lactate, Glucose, Glucose–lactate ratio, Prognosis, Mortality, Prediction, Secondary injury

## Abstract

**Background:**

Traumatic brain injury (TBI) is a major cause of trauma-related deaths. Systemic glucose and lactate levels reflect secondary metabolic derangements over time; however, most prognostic models rely on admission values. This study compared static and longitudinal indices of glucose, lactate, and their ratio in relation to ICU mortality.

**Methods:**

This retrospective single-center study analyzed 479 non-diabetic adult patients with TBI admitted to a German university ICU (2013–2023). After 1:2 severity-balanced, outcome-stratified propensity score matching, 229 patients (150 survivors, 79 non-survivors) were included. Indices comprised admission values, means, clearance, time-weighted averages, variability, and dysglycemic burden. Outcome was ICU mortality, assessed using regression, mixed-effects modeling, and ROC analysis.

**Results:**

Longitudinal indices showed stronger associations. Time-weighted average lactate was the best independent predictor (OR 14.70, 95% CI [5.41–39.98], *p* < 0.001, AUC 0.73). Time-weighted average glucose–lactate ratio also independently predicted ICU mortality (OR 0.75, 95% CI [0.66–0.86], *p* < 0.001). Non-survivors exhibited persistently higher glucose, lactate and lower ratios, with lactate significantly elevated on all first ten ICU days (all *p* < 0.05). Admission values, clearance and variability were not predictive after adjustment.

**Conclusions:**

In critically ill non-diabetic patients with TBI, longitudinal time-weighted average lactate significantly outperformed admission values and glucose metrics for predicting ICU mortality in a severity-balanced cohort; the glucose–lactate ratio was associated but did not surpass lactate. These findings underscore the importance of longitudinal monitoring and support prioritizing lactate in multiparametric prognostic model to account for secondary injuries. Prospective validation is warranted to confirm external validity and assess therapeutic implications.

**Supplementary Information:**

The online version contains supplementary material available at 10.1186/s40560-026-00864-9.

## Background

Traumatic brain injury (TBI) affects annually up to 70 million individuals worldwide and accounts for up to half of all trauma-related deaths [1, 2]. Beyond the initial insult, secondary injuries are major determinants of outcome and remain potential targets for intensive care management [3]. Early identification of patients at high risk of mortality is, therefore, critical [4].

Systemic metabolic dysregulation is a hallmark of secondary injury cascade after TBI. Experimental and clinical data have shown that post-traumatic stress response, inflammation, and mitochondrial dysfunction trigger disturbances in glucose and lactate metabolism [5]. Both metabolites are closely linked to the cerebral energy supply: glucose is the brain’s main substrate [6], whereas lactate, once considered a mere by-product of anaerobic metabolism [7], may also serve as an alternative fuel and signaling molecule [8–10].

Because direct measurement of cerebral concentrations requires invasive microdialysis, systemic blood levels have been investigated as surrogate markers [11, 12]. Previous studies reported prognostic associations for admission values [13–18], early maxima, clearance and mean concentrations [7, 14, 15, 19, 20] or glycemic variability [21, 22]; however, the findings were heterogeneous and cutoffs varied widely.

Prognostic models, such as the IMPACT–TBI model or models based on machine learning, primarily rely on demographic, clinical, and radiological variables, with glucose included only as a single admission value [23–25]. Although these models are widely used, they may fail to capture the evolving metabolic disturbances that characterize secondary injury processes after TBI.

We addressed this gap from an intensive care perspective by evaluating longitudinal, time-weighted metabolic indices that capture both the magnitude and duration of dysregulation. To isolate secondary injury-related metabolic signals beyond primary severity, we used propensity score matching for balancing primary severity. Within this prediction-focused cohort, we compared longitudinal and static glucose, lactate and glucose–lactate ratio indices and analyzed their trajectories over time to determine the best-performing measures for ICU mortality discrimination.

## Methods

### Study design

This retrospective, single-center observational study was conducted in accordance with the recommendations of the Ethics Committee of the Medical Faculty, RWTH Aachen University (EK 23-299). Given the retrospective and anonymized nature of the analysis, written patient consent was waived. All procedures adhered to the Declaration of Helsinki, institutional standards, and applicable guidelines. To enhance transparency, this study was reported following the STROBE (Strengthening the Reporting of Observational Studies in Epidemiology) guidelines [26]. The primary endpoint was ICU mortality, defined as death during ICU stay, irrespective of the cause.

### Patient selection

All patients admitted to the Department of Intensive Care Medicine, RWTH Aachen University Hospital, between July 2013 and October 2023 with TBI (ICD-10-GM S06) were screened. Exclusion criteria were: (i) absence of TBI, (ii) ICU stay < 72 h or withdrawal of life sustaining treatment within 72 h, (iii) < 4 blood gas analyses/day on average, (iv) age < 18 years, and (v) diabetes mellitus, given its pre-existing glycemic dysregulation and approximately 1.5-fold increase in TBI-related mortality [27, 28].

### Data collection

Clinical data were retrieved from electronic patient records (IntelliSpace Critical Care and Anesthesia, Koninklijke Philips N.V., Amsterdam, version J.05.00; Medico, CompuGroup Medical SE & Co. KGaA, Koblenz, version 29.00.02.00) and stored in Excel (Microsoft, Redmond, USA). Collected variables included (a) demographics: age, sex, body mass index (BMI); (b) injury severity/mechanism: glasgow coma scale (GCS) at admission, isolated TBI; (c) comorbidities: hypertension, peripheral arterial disease, chronic obstructive pulmonary disease (COPD), asthma bronchiale, chronic kidney disease, nicotine, alcohol or drug abuse; (d) ICU/hospital stay: simplified acute physiology (SAPS II) at admission, worst organ failure assessment (SOFA) score during ICU stay, length of ICU/hospital stay (LOS), duration of mechanical ventilation; (e) ICU complications: pneumonia, acute respiratory distress syndrome (ARDS), urinary tract infection, sepsis, septic shock, wound infection, acute kidney injury, delirium and (f) ICU mortality.

### Data extraction of blood gas analysis values

All arterial blood gas analyses (ABL90 FLEX, Radiometer, Krefeld, Germany) were extracted automatically via the institutional data warehouse (SAP SE, Walldorf, Germany, version 14.2.6.2953), including exact timestamps. Glucose was recorded in mg/dL and lactate in mmol/L; devices underwent routine calibration per manufacturer specifications. Data processing was performed using RStudio (Posit PBC, Boston, MA, USA, version 2025.09.0 + 387) with R version 4.5.1.

### Calculation of metabolic parameter indices

**Fig. 1:**
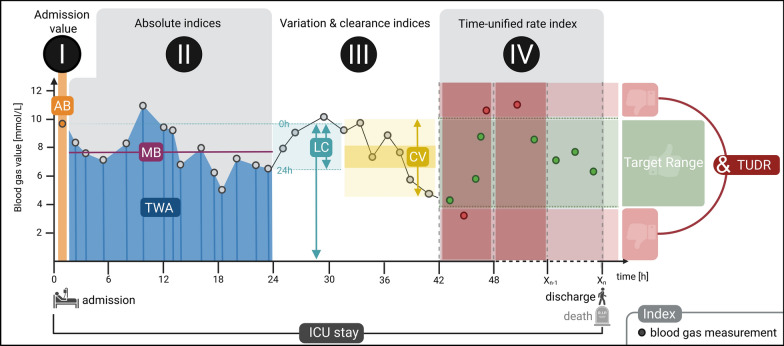
Calculation of metabolic marker indices. Calculation principles of the indices analyzed in this study are depicted here for a fictional patient example. Time between ICU admission and discharge is plotted on the *x*-axis and the measurement value on the *y*-axis. All indices of categories II–IV (except from lactate clearance) were calculated over the entire ICU stay but are shown here in a truncated segment for visual clarity. (**I**) Admission blood gas value (AB) was the first recorded blood gas value after ICU admission. (**II**) The absolute mean blood gas value (MB) represents the arithmetic mean of all measured values at a certain time. The time-weighted average (TWA) index represents the area under the curve formed by the blue trapezoids divided by the total observation time. (**III**) The coefficient of variation (CV) expresses the standard deviation relative to the mean, shown as a yellow ratio in the figure. Lactate clearance (LC) represents the percentage change over time (here 24 h LC) between the first and last values. (**IV**) The time-unified dysglycemic rate (TUDR) reflects the proportion of fixed-length intervals (e.g., 6 h) in which glucose values exceed the target range. In this example, two of the three intervals were outside this range, resulting in a TUDR of 66.7%. Created with BioRender. Deininger, M. (2025) https://BioRender.com/okax1gy; ICU intensive care unit

This study aimed to comprehensively characterize the metabolic markers glucose, lactate, and glucose–lactate ratio. Due to the retrospective design, the number and timing of arterial blood gas (ABG) measurements varied among patients. As these parameters are continuously influenced by dynamic physiological processes, temporal resolution is essential for meaningful interpretation. Relying solely on arithmetic mean values may bias results toward phases with frequent short-term sampling and obscure relevant trends. To account for this, four index categories were defined for each marker (Fig. 1):

I. Admission value (AB): First ABG measurement obtained after ICU admission, no later than 6 h post-admission.
II. Mean (MB) and time-weighted average (TWA): MB represents the arithmetic average of all measurements per patient. To incorporate the temporal distribution of values, TWA was calculated using the trapezoidal rule, where the area under the measurement curve is divided by the total observation time [29].
III. Variability (CV) and clearance (LC): Variability was expressed as coefficient of variation (CV), defined as standard deviation divided by the mean (MB), reported as percentage [30]. For lactate, clearance (LC) was calculated as percentage change from admission value at 6, 24 respective 72 h [31]. In addition to clearance based on the first and last observed raw values within the window (raw-LC), the 6/24/72 h lactate concentration was estimated by linear interpolation between the nearest measurements bracketing this endpoint to account for irregular sampling (ip-LC).
IV. Time-unified dysglycemic rate (TUDR): For blood glucose, the relative duration outside the target range (70–180 mg/dL) was calculated using the recently developed time-unified dysglycemic rate (TUDR). Detailed calculations were described elsewhere [32]. Briefly, each patient’s ICU stay was divided into equal-length periods starting with ICU admission. The shortest period ensuring ≥ 95% median coverage with at least one glucose value per period and patient was selected. The considered period lengths were 1, 2, 3, 4, 6, 8, and 12 h. TUDR was calculated as the number of periods outside the target range divided by all periods containing at least one measurement.

An overview of the indices analyzed for each metabolic marker is presented in Table 1. The time-weighted average absolute values per period were used to visualize the trend of metabolic parameters over time. In addition to indices over the entire ICU stay, TWA indices were also calculated over the first 72 h after ICU admission (72 h-TWAG, 72 h-TWAL, and 72 h-TWAGL) to reflect a pragmatic early prediction horizon. For the glucose–lactate ratio, glucose values were converted to mmol/L (mg/dL ÷ 18). It thus reflects a molar ratio defined as glucose [mmol/L]/lactate [mmol/L].

**Table 1:** Overview of the metabolic marker indices

Category	Index description	Metabolic marker indices		
		Glucose	Lactate	Glucose–lactate ratio
I	Value on ICU-admission (AB)	ABG (admission blood glucose)	ABL (admission blood lactate)	ABGL (admission blood glucose–lactate ratio)
II	Mean absolute index (MB)	MBG (mean blood glucose)	MBL (mean blood lactate)	MBGL (mean blood glucose–lactate ratio)
	Time-weighted average (TWA)	TWAG (time-weighted average glucose)	TWAL (time-weighted average lactate)	TWAGL (time-weighted average glucose–lactate ratio)
III	Coefficient of variation (CV)	CVG (coefficient of variation for glucose)	CVL (coefficient of variation for lactate)	CVGL (coefficient of variation for glucose–lactate ratio)
	Lactate clearance (LC)		LC (calculated for 6, 24 and 72 h)	
IV	Time-unified dysglycemic rate (TUDR)	TUDR (target range 70–180 mg/dL)		

### Standard treatment of TBI

All patients were treated according to the current national German guidelines for TBI [33]. Treatment aimed to prevent secondary cerebral injury and organ dysfunction through individualized control of intracranial pressure, cerebral perfusion, oxygenation, and systemic/metabolic homeostasis. Throughout the manuscript the term ‘secondary injury’ refers to secondary injury processes after TBI, including secondary cerebral as well as systemic complications or organ dysfunction. Blood glucose was managed by a nurse-driven protocol. Continuous intravenous insulin infusion was initiated for persistent hyperglycemia exceeding 180 mg/dL, while in case of hypoglycemia (< 70 mg/dL), glucose was administered in accordance with international intensive care guidelines [34].

### Disclosure of AI usage

During the preparation of this manuscript, the authors used AI tools, including Paperpal by Editage (version 4.16.4) and ChatGPT by OpenAI (version ChatGPT-5 Thinking/Pro) to improve language and readability. All outputs were reviewed, adapted, and integrated into the final work by the authors, who take full responsibility for all content presented herein.

### Statistical analysis

Statistical analyses were performed with SPSS (IBM, version 29.0.2.0(20)) and RStudio (Posit PBC, Boston, MA, USA, version 2025.09.0 + 387) with R (version 4.5.1). All tests were two-tailed, and *p* values < 0.050 were considered statistically significant.

Propensity score matching (PSM) was used to create a baseline-severity-balanced, outcome-stratified cohort, reducing confounding for association analyses and subsequent multivariable modeling within the matched sample. No causal effects were estimated or inferred. Propensity scores were calculated with logistic regression including only pre-exposure variables available at ICU admission using the MatchIt R-package. A 1:2 nearest-neighbor matching without replacement was performed using a caliper of 0.2 standard deviations of the logit propensity score, including as covariates: age, sex, BMI, GCS, isolated TBI, hypertension, chronic kidney disease, and SAPS II score. Common support was verified using propensity score overlap diagnostics and the exclusion of unmatched cases outside the caliper. Matching performance was assessed using standardized mean differences (SMD) with a target threshold of < 0.10; *p* values are reported descriptively and were not used to judge balance. Covariate balance before and after matching was visualized using a Love plot (R-package cobalt).

Data were analyzed with Mann–Whitney *U* test and categorical variables with Fisher’s exact test. A linear mixed-effects model with repeated measures was used to assess the association between metabolic parameters and ICU mortality. The model included fixed effects for survival, time, and their interactions to assess group-specific time trends. A random intercept for each patient was included to account for the within-subject correlation. The repeated structure was modeled using an autoregressive covariance structure of the first order. To account for nonlinear trends, time was modeled using restricted cubic splines (RCS) with five degrees of freedom. The number of knots was selected based on likelihood ratio tests and comparisons of Akaike Information Criterion values. For statistical modeling, daily TWA values (24 h periods) were used to ensure robustness and interpretability, whereas 6 h intervals were applied for RCS-based analysis and visualization to capture a finer temporal resolution. Estimated marginal means with 95% confidence intervals (95% CI) were computed for each group and ICU day based on fitted models. Bonferroni-adjusted pairwise contrasts were used to test daily differences between survivors and non-survivors. Results were visualized using groupwise plots of predicted means with 95% CI. Receiver operating characteristic (ROC) curve analysis was performed to evaluate the predictive performance of the metabolic indices for survival. The area under the curve (AUC) and corresponding 95% CI were calculated to quantify discriminative ability. Multivariable logistic regression analysis was performed to examine the effects of metabolic markers, considering possible confounders. Multicollinearity in multivariable models was evaluated using the variance inflation factor (VIF). However, first, to assess multicollinearity between predictors, pairwise Spearman correlation coefficients were calculated; a correlation coefficient above 0.50 was considered indicative of high collinearity. If various indices showed multicollinearity, the time-weighted model was preferentially used for subsequent analyses; if necessary, separate multivariable models were specified. Candidate predictors were chosen a priori and supplemented by univariable screening, balancing clinical plausibility with events-per-variable considerations. Standard errors were estimated with cluster-robust variance at the match-set level, and a conditional logistic regression stratified by match-set as well as conventional logistic regression with classical (Wald) served as a sensitivity analysis and were included in the Supplement. Odds ratios (OR) with 95% CI and for continuous predictors, *z*-scores were reported. The predictive performance of multivariable-significant indices was subsequently compared using the DeLong test for paired ROC curves (R-package, pROC). A supplementary robustness analysis examined whether the index with the greatest impact could be explained solely by systemic complications. Therefore, the logistic regression model was expanded by significant between-group complications to perform a post-baseline sensitivity analysis. To assess the influence of extracranial disease severity, sensitivity analyses were performed using cluster-robust variance multivariable logistic regression, which included non-isolated TBI as an additional parameter, injury severity score (ISS) in non-isolated TBI patients and admission non-neurological SOFA (non-c-SOFA) instead of admission SAPS II. Details on the calculation of non-c-SOFA can be found elsewhere [35]. The calculation is identical to the conventional SOFA score, including worst values within first 24 h, excluding the neurological (GCS) component.

**Fig. 2:**
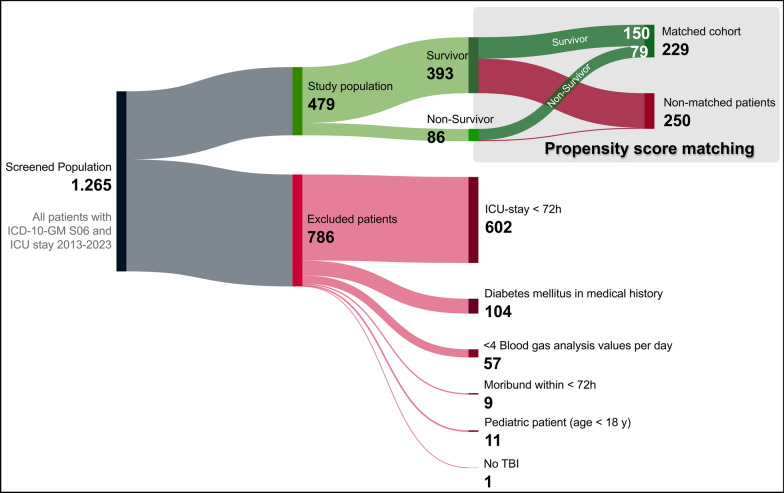
Study population flow chart. *GM* german modification; *ICD* international classification of diseases; *ICU* intensive care unit; *TBI* traumatic brain injury

**Fig. 3:**
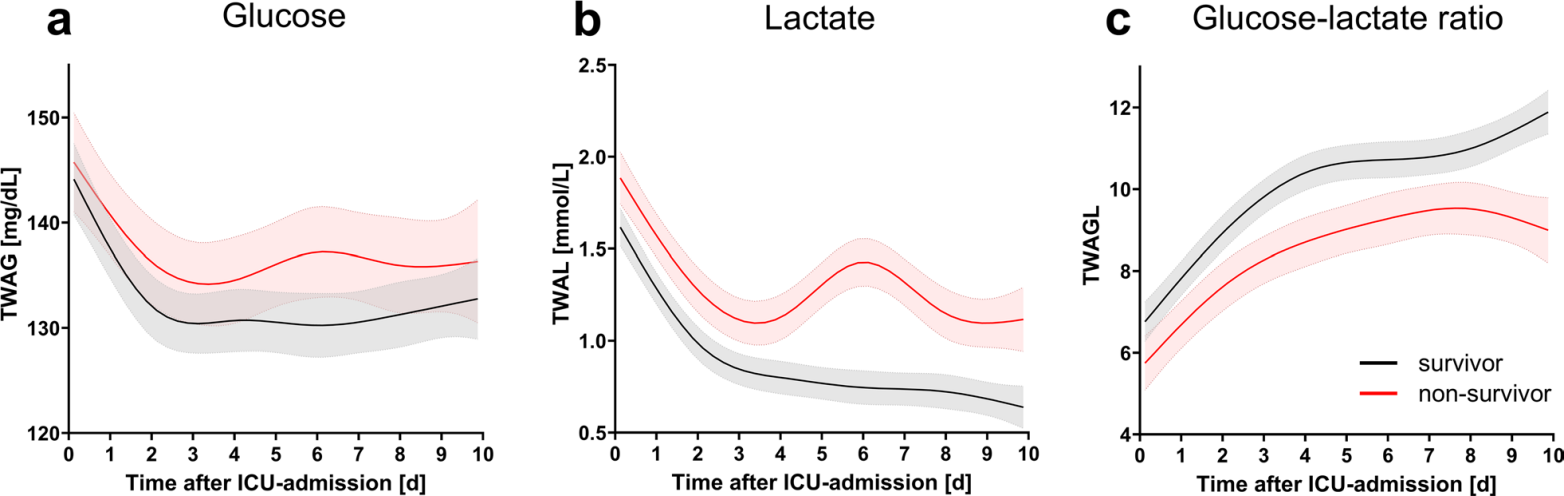
Trend of glucose and lactate over ICU stay. The figure shows the trend over time of (**a**) glucose (TWAG), (**b**) lactate (TWAL), and (**c**) glucose–lactate ratio (TWAGL) over the first ten ICU days, stratified by survival status. The time is shown in days on the *x*-axis and the corresponding measured value on the *y*-axis. The 6 h-time-weighted values form the database (raw value curves are depicted in Figure S2, Supplement), calculated estimated marginal mean intervals derived from restricted cubic spline mixed model with five degrees of freedom are shown here. Shaded ribbons represent 95% CI. *ICU* intensive care unit; *mg/dL* milligram per deciliter; *mmol/L* millimole per liter; *TWAG* time-weighted average glucose; *TWAGL* time-weighted average glucose–lactate ratio; *TWAL* time-weighted average lactate

**Fig. 4:**
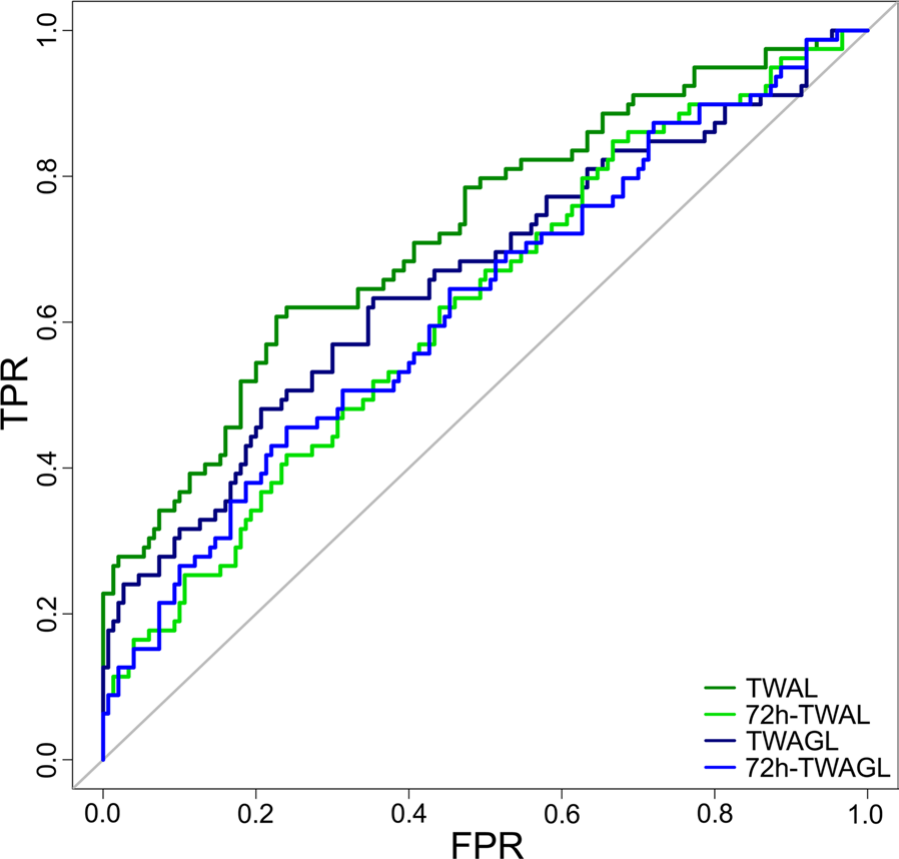
ROC curves for lactate and glucose–lactate indices. A comparative depiction of the four receiver operating characteristic (ROC) curves for all significant indices of the respective multivariable analyses for mortality prediction are shown. Time-weighted average curves for lactate (TWAL) are shown in green, for the glucose–lactate ratio (TWAGL) in blue, with the lighter color representing the index of the first 72 h each. *TWAGL* time-weighted average glucose–lactate ratio; *TWAL* time-weighted average lactate, *TPR* true positive rate, *FPR* false positive rate

Data are presented as median (25. to 75. interquartile range, IQR), absolute numbers (frequencies), or OR with 95% CI. Figure 1 was created using BioRender.com. Figure 2 was created using SankeyMATIC (Sankeymatic.com) and Adobe Photoshop CS 6 (Adobe Systems Software Ireland Limited, Dublin, Ireland; version 13.0). Figures 3 and S3 were generated using Prism (GraphPad Software Inc., San Diego, USA, version 10.6.1 (892)). Figure 4 was created using R-package pROC.

## Results

### Study population and patient characteristics

All patients admitted to the Department of Intensive Care Medicine of RWTH Aachen University Hospital between July 2013 and October 2023 were screened for traumatic brain injury. Following in- and exclusion criteria, 479 of the initially screened 1265 ICU patients were included in the study (Fig. 2). More than four fifths (*n* = 393; 82%) survived the initial ICU stay (survivor group), the remaining 18% (*n* = 86) were assigned to the non-survivor group.

Demographic data, especially age, GCS, isolated TBI, hypertension, chronic kidney disease, and SAPS II score, showed significant group differences; hence, 1:2 propensity score matching (PSM) was performed to achieve the best possible severity-balanced group comparability (Table 2).

**Table 2:** Patient characteristics

*Study Population*							
	Cohort	Pre-PSM			Post-PSM (= matched cohort)		
	*n* = 479	Survivor *n* = 393 (82.0%)	Non-survivor *n* = 86 (18.0%)	*p* value	Survivor *n* = 150	Non-survivor *n* = 79	*p* value
Age [years]	63 (43–77)	61 (40–75)	74 (56–83)	** < 0.001**	70 (56–78)	73 (55–82)	0.554
Sex (male)	331 (69.1%)	268 (68.2%)	63 (73.3%)	0.440	106 (70.7%)	59 (74.7%)	0.540
BMI [kg/m^2^]	24.9 (23.1–27.8)	25.0 (23.1–27.8)	24.7 (23.1–27.5)	0.202	25.4 (23.3–27.7)	24.7 (23.4–27.7)	0.376
GCS	9 (5–13)	9 (5–13)	8 (3–12)	**0.040**	8 (4–12)	9 (3–12)	0.992
Isolated TBI	216 (45.1%)	168 (42.7%)	48 (55.8%)	**0.031**	81 (54.0%)	42 (53.2%)	1.000
*Medical History*							
Hypertension	171 (35.7%)	132 (33.6%)	39 (45.3%)	**0.047**	64 (42.7%)	34 (43.0%)	1.000
Peripheral arterial disease	18 (3.8%)	15 (3.8%)	3 (3.5%)	1.000	9 (6.0%)	2 (2.5%)	0.338
COPD	26 (5.4%)	19 (4.8%)	7 (8.1%)	0.289	15 (10.0%)	6 (7.6%)	0.636
Asthma bronchiale	11 (2.3%)	10 (2.5%)	1 (1.2%)	0.698	2 (1.3%)	1 (1.3%)	1.000
Chronic kidney disease	26 (5.4%)	14 (3.6%)	12 (14.0%)	**< 0.001**	11 (7.3%)	9 (11.4%)	0.330
Nicotine abuse	45 (9.4%)	37 (9.4%)	8 (9.3%)	1.000	17 (11.3%)	8 (10.1%)	0.828
Alcohol abuse	67 (14.0%)	57 (14.5%)	10 (11.6%)	0.607	17 (11.3%)	10 (12.7%)	0.830
Drug abuse	28 (5.8%)	26 (6.6%)	2 (2.3%)	0.200	7 (4.7%)	2 (2.5%)	0.722
*Scores*							
SAPS II score	35 (28–41)	34 (27–40)	40 (32–45)	**< 0.001**	38 (23–43)	40 (30–43)	0.706
Worst SOFA score^a^	10 (8–13)	10 (8–12)	12 (10–13)	**< 0.001**	10 (8–12)	12 (10–13)	**< 0.001**
*Complications*							
Pneumonia	192 (40.1%)	159 (40.5%)	33 (38.4%)	0.808	58 (38.7%)	29 (36.7%)	0.886
ARDS	20 (4.2%)	16 (4.1%)	4 (4.7%)	0.768	3 (2.0%)	4 (5.1%)	0.238
Urinary tract infection	36 (7.5%)	32 (8.1%)	4 (4.7%)	0.367	11 (7.3%)	4 (5.1%)	0.587
Sepsis	77 (16.1%)	55 (14.0%)	22 (25.6%)	**0.014**	23 (15.3%)	19 (24.1%)	0.110
Septic shock	51 (10.6%)	34 (8.7%)	17 (19.8%)	**0.006**	15 (10.0%)	16 (20.3%)	**0.041**
Wound infection	21 (4.4%)	19 (4.8%)	2 (2.3%)	0.395	5 (3.3%)	2 (2.5%)	1.000
Acute kidney injury	39 (8.1%)	21 (5.3%)	18 (20.9%)	**< 0.001**	9 (6.0%)	17 (21.5%)	**< 0.001**
Delirium	77 (16.1%)	75 (19.1%)	2 (2.3%)	**< 0.001**	27 (18.0%)	2 (2.5%)	**< 0.001**
*Outcomes*							
ICU–LOS [days]	13.8 (6.7–28.2)	15.1 (7.4–30.8)	8.8 (5.5–18.0)	**< 0.001**	15.0 (7.2–30.1)	8.9 (5.5–17.6)	**0.002**
Hospital LOS [days]	19.0 (10.0–33.0)	23.0 (11.0–35.0)	10.0 (6.0–18.3)	**< 0.001**	22.0 (10.0–35.0)	10.0 (6.0–18.0)	**< 0.001**
Mechanical ventilation [hours]	242 (46–497)	257 (30–514)	175 (115–340)	0.839	252 (49–502)	175 (116–337)	0.959

Data are presented as median (IQR) or absolute number (percentage in the group). Significant *p* values are shown in bold

*ARDS* acute respiratory distress syndrome; *BMI* body mass index; *COPD* chronic obstructive pulmonary disease; *GCS* glasgow coma scale; *ICU* intensive care unit; *kg/m*^*2*^ kilogram per square meter; *LOS* length of stay; *PSM* propensity score matching; *SAPS II* simplified acute physiology score II; *SOFA* sequential organ failure assessment score; *TBI* traumatic brain injury

^a^ Worst SOFA is defined as maximum SOFA during ICU stay; as a post-admission value it was not used for PSM

Reasons for non-matching were primarily lack of suitable controls within the caliper and out-of-support propensity scores. PSM showed a reduction in the standardized mean differences to < 0.10 for all covariates (Supplement Figure S1). The matched cohort post-PSM included 150 patients in the survivor and 79 in the non-survivor group. No significant group differences were found post-PSM for demographic data, medical history, GCS, SAPS II score on admission, the proportion of isolated TBI and the time to first blood gas analysis after ICU admission (non-survivor: 0.25 (0.16–0.50) h; survivor: 0.27 (0.16–0.60) h, *p* = 0.758). Non-survivors had a significantly higher rate of septic shock (non-survivor: 20.3%; survivor: 10.0%, *p* = 0.041), acute kidney injury (non-survivor: 21.5%; survivor: 6.0%, *p* < 0.001) and consequently a higher worst SOFA score (non-survivor: 12 (10–13); survivor: 10 (8–12), *p* < 0.001). In addition, non-survivors had in median about 6 days shorter ICU as well as 12 days shorter hospital length of stay (*p* < 0.001) and an about seven times lower delirium rate (*p* < 0.001) than the survivor group. No significant group differences were observed for any of the other complications examined.

### Survivors show decreased glucose and lactate values over time

Figure 3 illustrates the course of glucose, lactate and their ratio over the first ten ICU days, stratified by survival status (raw data curves in Supplement Figure S2). Based on linear mixed-effects model, all three metabolic marker indices showed significantly different values in non-survivors across the entire ICU stay (TWAG: *p* = 0.036; TWAL: *p* < 0.001; TWAGL: *p* < 0.001). Pairwise comparisons revealed daily group differences in lactate, glucose on day 6, and glucose–lactate ratio on days 3–6 and 8–10 after Bonferroni correction. Spline-based modeling showed significant nonlinearity in the parameter kinetics for all three indices (all *p* < 0.001). Moreover, survival status significantly modified the temporal course of lactate (*p* = 0.006) and glucose–lactate ratio (*p* = 0.016), but not glucose. Glucose levels declined over the first 3 days, with a discreet, transient rise in non-survivors on days 6–7, whereas the glucose–lactate ratio increased steadily until day 8, with a reduced slope from day 3, after which a decrease was observed in non-survivors. In contrast, lactate levels in survivors declined rapidly during the first 3 days and then nearly plateaued, whereas non-survivors exhibited a biphasic pattern with an initial decrease up to day 3 followed by a secondary peak from days 4 to 8.

### Non-survivors showed elevated metabolic indices

As lactate and glucose showed rapid decreasing and their ratio increasing trend over the first 72 h of ICU stay, the absolute average indices (II) for this period were calculated in addition to the values for the entire ICU stay. Compared to survivors, non-survivors showed significantly higher values for both blood glucose and lactate (Table 3). However, the variability over time and lactate clearance were not significantly different, and the admission blood value was significantly divergent only for glucose, but not for lactate. In contrast, for the first 72 h, the time-weighted average value for lactate was significantly higher in the non-survivor group, but not for glucose. The mean glucose–lactate ratio was significantly lower in the non-survivor compared to the survivor group (non-survivor: 7.9 (6.5–10.1); survivor: 9.7 (8.1–11.1), *p* < 0.001), and time-weighted average values over the entire ICU stay and first 72 h yielded analogous results. In contrast to glucose and lactate, when analyzed individually, a significantly higher variability of the glucose–lactate ratio was observed for the non-survivors compared to survivors (non-survivor: 34.5 (25.2–42.2); survivor: 30.1 (24.5–36.4), *p* = 0.016).

**Table 3:** Glycemic, lactate and glucose–lactate ratio indices

	Survivor n = *150*	Non-survivor n = *79*	*p *value
Blood glucose			
ABG [mg/dL]	140.0 (115.0–161.0)	154.0 (131.0–175.0)	**0.005**
MBG [mg/dL]	129.8 (122.2–139.6)	134.1 (123.7–147.3)	**0.031**
72 h-TWAG [mg/dL]	134.4 (124.2–147.3)	138.1 (125.4–154.6)	0.236
TWAG [mg/dL]	127.9 (119.7–138.4)	134.5 (123.3–147.6)	**0.015**
CVG [%]	16.5 (13.9–19.0)	16.9 (14.5–20.9)	0.096
TUDR [%]	3.2 (0.0–8.9)	8.0 (3.9–15.4)	** < 0.001**
Lactate			
ABL [mmol/L]	1.60 (0.90–2.30)	1.50 (1.00–2.60)	0.172
MBL [mmol/L]	0.85 (0.71–1.06)	1.12 (0.91–1.57)	** < 0.001**
72 h-TWAL [mmol/L]	1.04 (0.81–1.45)	1.30 (0.96–1.73)	**0.002**
TWAL [mmol/L]	0.84 (0.72–0.99)	1.08 (0.86–1.45)	** < 0.001**
CVL [%]	41.9 (32.1–58.0)	44.8 (31.8–63.8)	0.681
72 h-ip-LC^a^ [%]	50.0 (16.1–68.4)	40.2 (6.5–68.2)	0.435
Glucose–lactate ratio			
ABGL	5.1 (3.5–8.7)	5.6 (3.3–7.8)	0.521
MBGL	9.7 (8.1–11.1)	7.9 (6.5–10.1)	** < 0.001**
72 h-TWAGL	8.1 (6.5–10.1)	6.9 (5.2–9.0)	**0.002**
TWAGL	9.5 (8.1–11.0)	8.2 (6.6–10.1)	** < 0.001**
CVGL [%]	30.1 (24.5–36.4)	34.5 (25.2–42.2)	**0.016**

Data are presented as median (IQR). Significant *p* values are shown in bold

*ABG* admission blood glucose; *ABGL* admission blood glucose–lactate ratio; *ABL* admission blood lactate; *CVG* coefficient of variation for glucose; *CVGL* coefficient of variation for glucose–lactate ratio; *CVL* coefficient of variation for lactate; *ip-LC* interpolated lactate clearance; *MBG* mean blood glucose; *MBGL* mean blood glucose–lactate ratio; *MBL* mean blood lactate; *mg/dL* milligram per deciliter; *mmol/L* millimole per liter; *TUDR* time-unified dysglycemic rate; *TWAG* time-weighted average glucose; *TWAGL* time-weighted average glucose–lactate ratio; *TWAL* time-weighted average lactate

^a ^For better readability, all ip-LC and raw-LC values are shown in Supplement Table S1. For all three LC time periods (6, 24, 72 h), there were no statistically significant group differences

### Lactate showed superior predictive value compared to glucose

To evaluate the predictive accuracy of the two metabolic markers, glucose, lactate, and their ratio for ICU mortality, the AUC was analyzed (Table 4). Lactate indices showed highest (TWAL/MBL: 0.73; 72 h-TWAL: 0.62), glucose–lactate ratio (MBGL: 0.69; TWAGL: 0.66; 72 h-TWAGL: 0.63) moderate and glucose indices (MBG: 0.59; TWAG: 0.60) weakest discrimination potential.

**Table 4:** Area under the curve for non-survival

Variable	AUC [95% CI]
ABG [mg/dL]	0.61 [0.54–0.69]
MBG [mg/dL]	0.59 [0.51–0.67]
TWAG [mg/dL]	0.60 [0.52–0.68]
TUDR [%]	0.67 [0.59–0.74]
MBL [mmol/L]	0.73 [0.66–0.80]
72 h-TWAL [mmol/L]	0.62 [0.55–0.70]
TWAL [mmol/L]	0.73 [0.66–0.80]
MBGL*	0.69 [0.61–0.76]
72 h-TWAGL*	0.63 [0.55–0.70]
TWAGL*	0.66 [0.59–0.74]
CVGL [%]	0.60 [0.52–0.68]

*Predictive value for survival

*ABG* admission blood glucose; *AUC* area under the curve; *CVGL* coefficient of variation for glucose–lactate ratio; *MBG* mean blood glucose; *MBGL* mean blood glucose–lactate ratio; *MBL* mean blood lactate; *mg/dL* milligram per deciliter; *mmol/L* millimole per liter; *TUDR* time-unified dysglycemic rate; *TWAG* time-weighted average glucose; *TWAGL* time-weighted average glucose–lactate ratio; *TWAL* time-weighted average lactate

### Lactate showed highest predictive value

Multivariable logistic regression was performed to examine the predictive value of these factors, considering confounders. Based on the Spearman test ABG, TWAG, TWAL, and TWAGL were included for the entire ICU stay and for the analysis of the first 72 h 72 h-TWAL and 72 h-TWAGL. After collinearity between lactate and glucose–lactate-ratio indices were found (TWAL–TWAGL: 0.87, 72 h-TWAL–72 h-TWAGL: 0.92; Supplement Figure S3), two separate multivariable logistic regression analyses were performed for each time period. The first examined glucose and lactate indices (ABG, TWAG, [72 h-]TWAL) and the second examined the glucose–lactate-ratio index ([72 h-]TWAGL) along with demographic aspects. The results were then ranked. Univariable logistic regression identified ABG, TWAG, [72 h-]TWAL, [72 h-]TWAGL as possible predictors of ICU mortality (Table 5). Furthermore, SAPS II score was considered a relevant mortality-associated demographic variable and hence included. Across these four multivariable models, all VIF values were ≤ 1.3, indicating no noteworthy collinearity (Supplement Table S2).

**Table 5:** Regression analysis

Variables	Univariable regression		Multivariable regression							
			Glucose and lactate				Glucose–lactate ratio			
			First 72 h		Full ICU stay		First 72 h		Full ICU stay	
	OR [95% CI]	*p* value	OR [95% CI]	*p* value	OR [95% CI]	*p* value	OR [95% CI]	*p* value	OR [95% CI]	*p* value
Age [years]	0.998 [0.984–1.012]	0.777								
Sex (male)	0.817 [0.441–1.514]	0.520	0.896 [0.428–1.875]	0.770	1.003 [0.448–2.247]	0.994	0.944 [0.454–1.964]	0.878	0.910 [0.435–1.904]	0.803
BMI [kg/m^2^]	0.979 [0.913–1.050]	0.553								
GCS	1.003 [0.938–1.072]	0.932								
Isolated TBI	1.034 [0.599–1.786]	0.904								
Hypertension	1.015 [0.585–1.761]	0.957								
CKD	1.625 [0.643–4.104]	0.305								
SAPS II score	1.004 [0.974–1.034]	0.807	1.000 [0.977–1.023]	0.997	0.991 [0.964–1.018]	0.514	1.004 [0.982–1.027]	0.715	1.000 [0.977–1.024]	0.975
ABG	1.008 [1.001–1.015]	**0.023**	1.005 [0.998–1.013]	0.155	1.003 [0.993–1.013]	0.589				
TWAG	1.024 [1.007–1.042]	**0.006**			1.015 [0.992–1.039]	0.197				
TWAL	18.609 [6.036–57.373]	**< 0.001**			14.701 [5.406–39.976]	**<0.001**				
72 h-TWAL	2.358 [1.429–3.893]	**< 0.001**	2.127 [1.337–3.384]	**< 0.001**						
TWAGL	0.752 [0.660–0.857]	**< 0.001**							0.754 [0.660–0.860]	**<0.001**
72 h-TWAGL	0.834 [0.745–0.932]	**< 0.001**					0.835 [0.742–0.939]	**0.003**		

Multivariable logistic regression models were adjusted for baseline covariates. Odds ratios were reported separately for the two analyzed time horizons (first 72 h and full ICU stay). Only non-redundant indices were used to minimize collinearity. Significant *p* values are shown in bold

*ABG* admission blood glucose; *BMI* body mass index; *CKD* chronic kidney disease; *GCS* glasgow coma scale; *kg/m*^*2*^ kilogram per square meter; *LOS* length of stay; *SAPS II* simplified acute physiology score II; *TBI* traumatic brain injury; *TWAG* time-weighted average glucose; *TWAGL* time-weighted average glucose–lactate ratio; *TWAL* time-weighted average lactate

The multivariable logistic regressions analyzing glucose and lactate revealed a significant mortality prediction for the lactate index TWAL with an odds ratio of 14.701 (95% CI 5.406–39.976, *p* < 0.001, *z*-score: 5.266) as well as 72 h-TWAL with an odds ratio of 2.127 (95% CI 1.337–3.384, *p* < 0.001, *z*-score: 3.188). No significant results were observed for the two glucose indices, TWAG and ABG. The multivariable logistic regression including glucose–lactate ratios showed a significant mortality protection prediction value at higher TWAGL ratios (72 h-TWAGL: OR 0.835, 95% CI [0.742–0.939], *p* = 0.003, *z*-score: -3.001; TWAGL: OR 0.754, 95% CI [0.660–0.860], *p* < 0.001, *z*-score: − 4.202). Conditional and conventional logistic regressions showed comparable results (Supplement Tables S3, S4). The predictive accuracies of the significant indices from the multivariable models were compared using paired DeLong tests (Fig. 4).

The lactate index TWAL showed the highest discrimination and was significantly superior to the other three indices (TWAGL, 72 h-TWAGL, 72 h-TWAL; DeLong *p* values: 0.003, < 0.001, and 0.001, respectively), whereas no significant differences were observed among the latter three. In a robustness analysis including post-baseline adjustments for septic shock and acute kidney injury TWAL remained independently associated with ICU mortality (OR 12.204, 95% CI [4.500–33.097], *p* < 0.001, *z*-score: 4.915; Supplement Table S5). Sensitivity analyses assessing influence of extracranial disease severity showed that adding isolated TBI as a covariate (Supplement Table S6), replacing SAPS II with day 1 non-neurological SOFA (Supplement Table S7) or including ISS for non-isolated TBI patients yielded similar results (Supplement Table S8).

## Discussion

In this severity-balanced cohort of non-diabetic patients with TBI, we systematically compared systemic indices of glucose, lactate, and their ratio in relation to ICU mortality. The analysis demonstrated that time-weighted average lactate indices consistently provided a superior predictive value compared to glucose metrics. Glucose–lactate ratio, while associated, added limited incremental value and should only be considered complementary. In contrast, single admission, average, variance or clearance indices of either glucose or lactate were not reliable, underscoring the importance of longitudinal, dynamic indices over static measurements when assessing mortality after TBI.

### Lactate outperforms glucose

Our results confirm and extend prior reports that systemic lactate is a stronger prognostic marker than glucose in critically ill patients with TBI [14, 36, 37]. In our cohort, time-weighted average lactate values across the ICU stay and subordinately over the first 72 h were robustly associated with ICU mortality, whereas admission lactate value and lactate clearance were not. This supports the concept that sustained lactate elevation reflects ongoing metabolic stress and impaired clearance rather than a single acute event [38, 39]. The pathophysiological plausibility is strong: TBI induces a hypermetabolic state, driven by both systemic and cerebral inflammatory response, with up to 70% increased systemic lactate liberation and up to fourfold increased hepatic and renal gluconeogenesis [10, 38]. Consistently, time-weighted average lactate remained associated with ICU mortality after adjusting for injury pattern and severity, early extracranial dysfunction, as well as septic shock and acute kidney injury—indicating that systemic processes are a composition, not the sole origin, of the risk signal captured by persistent lactate elevation. Thus, persistent lactate elevation may reflect both secondary cerebral injury processes and systemic organ dysfunction or inflammation. Importantly, systemic and cerebral lactate concentrations are positively correlated [9], whereas glucose levels show inconsistent coupling [40]. This dual role of lactate, as energy substrate via the “lactate shuttle” and as a signaling molecule influencing inflammation, vascular tone, and intracranial pressure, is consistent with its stronger prognostic associations observed here, although mechanistic explanations remain speculative [8, 10].

### Glucose–lactate ratio as integrative marker

Beyond absolute concentrations, the glucose–lactate ratio may serve as a complementary composite index; however, in our cohort, its discrimination did not reach that of lactate. To our knowledge, this is the first study to evaluate the systemic glucose–lactate ratio in TBI. To calculate referential values, data from a published study comparing patients with TBI with healthy controls were used. Based on these data, ex-post calculated glucose–lactate ratio was 12.3 for healthy controls and 8.7 for trauma patients over the first 9 days [41]. In our data, the overall median was 9.0; survivors approached 12.3 by ICU day 10, whereas the median ratio of 9.0 was reached by non-survivors on day 7 but not exceeded during the observation period. These dynamics suggest that glucose–lactate ratio captures the balance between energy substrate availability and stress-related lactate production. However, given the superiority of and collinearity with lactate in terms of mortality prediction, the added prognostic value of glucose–lactate ratio for ICU mortality should be considered limited. Overall, the glucose–lactate ratio appears complementary rather than substitutive: it might be helpful for pathophysiological interpretation when glucose and lactate differ, but for predicting mortality, lactate burden seems to be dominant.

### Admission values, clearance and variability indices are insufficient

Neither admission glucose nor lactate reliably predicted outcome. Some prior studies found associations, others did not [13, 14, 17, 31, 37, 42, 43]. This inconsistency likely reflects heterogeneity in sampling context and timing (prehospital, emergency department, ICU), measurement variability or errors, and patient characteristics. Similarly, no significant group differences were observed in glucose or lactate variability. An effect was demonstrated only for the glucose–lactate ratio; however, there was considerable overlap between survivors and non-survivors, suggesting that variability alone may not be robust enough for clinical application. In contrast, an MIMIC-IV study reported the predictive value for glycemic variability but used a variability cutoff for grouping and analyzed a less severe cohort with shorter ICU stays (median admission GCS 13, ICU–LOS 3 days), limiting comparability [21]. Regarding lactate clearance, the evidence for mortality prediction after TBI is weak. The studies showed, consistent with the results of this study, that lactate clearance has no benefit compared to admission lactate [7]. These findings caution against overinterpretation of single admission values, short-term clearance or variability and argue for trend-based indices in clinical risk stratification.

### Glucose and lactate dynamics over time

Previous studies demonstrated early peaks in blood glucose and lactate after TBI followed by gradual normalization [44–48]. Consistent with this, our survivors exhibited initial elevations with subsequent recovery, whereas non-survivors maintained persistently high levels. Interestingly, we observed a secondary rise in both glucose and lactate around ICU days 5–6 among non-survivors, possibly reflecting delayed systemic or cerebral deterioration, as noted in earlier studies [44]. These kinetic patterns underscore the value of longitudinal rather than static assessments. Despite the second peak of glucose and lactate, their ratio remained relatively stable and may provide a complementary descriptor to contextualize proportional from disproportional changes over time.

### Clinical and therapeutic implications

From a clinical perspective, time-weighted average lactate could be integrated into multiparametric scores to refine risk stratification for secondary injuries. The glucose–lactate ratio may be considered supplementary when the interaction between glucose and lactate seems relevant. Supporting this, a recently published retrospective study demonstrated superiority of a modified IMPACT–TBI prediction model when including median lab values over the first 14 days after ICU admission instead of admission values inter alia for blood glucose [49]. Incorporating longitudinal, dynamic indices—particularly time-weighted average lactate—into prediction models might improve their calibration and discriminatory performance, especially when admission signals are equivocal. While full-stay indices yielded the best discrimination, first 72 h values may represent a pragmatic compromise between accuracy and timelines. With growing use of electronic health records, real-time calculation of such indices is technically feasible and could enable dynamic, individualized risk assessment and decision support at the bedside.

Therapeutically, both hypo- and hyperglycemia should be avoided as these have been associated with adverse outcomes [6], although strict intensive glucose control (80–110/120 mg/dL) has not demonstrated survival benefit over conventional management [50]. The role of lactate is more complex: while high endogenous levels indicate severe injury, exogenous lactate supplementation has paradoxically improved tissue energetics in some trials [51, 52]. These associations could be biologically plausible in the context of secondary injury cascades after TBI, including inflammation, mitochondrial dysfunction, and altered systemic clearance mechanisms; however, mechanistic interpretations remain speculative and beyond the scope of this retrospective analysis. Whether simultaneous modulation of glucose and lactate can influence outcome remains unclear but should be explored in future interventional studies.

### Strengths and limitations

A key strength of this study is the side-by-side evaluation of multiple glucose–, lactate– and glucose–lactate-ratio-based indices within a single severity-balanced cohort using propensity score matching, combined with frequent sampling enabling kinetic analyses.

However, limitations must be considered. The retrospective, single-center design and exclusion of diabetic patients reduce generalizability. Excluding patients with ICU stay < 72 h may have introduced survivorship bias, potentially underestimating metabolic extremes. This restriction was chosen to ensure sufficient temporal resolution of longitudinal metabolic indices, which require repeated measurements over several days. Findings should, therefore, be interpreted as primarily representative for patients with prolonged ICU treatment. As worst SOFA score differed between groups, it cannot be ruled out that differences in evolving organ dysfunction during ICU stay affected the reported associations, limiting strict pathophysiological interpretation. Representing routine ICU care, our cohort included both isolated and non-isolated TBI, which may blur pathophysiologic attribution; however, sensitivity analyses were performed to address this, including adjustment for injury pattern, organ dysfunction and extracranial injury severity. Sampling frequency for glucose and lactate was clinically driven and, therefore, variable; TWA/TUDR mitigated but did not eliminate frequency‑related bias. PSM restricts inference to common support, limiting generalizability to patients resembling the matched cohort. As non-causal, results reflect associations and discrimination, not treatment effects. This framing was chosen, as it prioritized clinically useful risk stratification under comparable primary severity. Pharmacological and nutritional influences (e.g., insulin, vasopressors and feeding) were not systematically controlled. The 10-year observation period introduced treatment-related heterogeneity. Absence of cerebral microdialysis precluded direct assessment of systemic-cerebral coupling. Finally, our outcome measure was limited to ICU mortality; functional neurological outcomes were not available.

Given these limitations, our findings should be interpreted as exploratory and hypothesis-generating only. Prospective multicenter studies with standardized protocols are needed to enhance external validity. Such designs would help validate the prognostic role of lactate and supplementary glucose–lactate ratio across diverse patient populations and care settings.

## Conclusions

Longitudinal time-weighted average lactate index was strongly associated with ICU mortality in a severity-balanced cohort of patients with TBI, outperforming admission values and glucose metrics. The composite time-weighted average glucose–lactate ratio was associated but did not surpass lactate and is best viewed as complementary. These findings emphasize the value of longitudinal monitoring and support prioritizing systemic lactate as practical component of future multimodal ICU prognostic models to account for secondary injuries. Prospective multicenter validation studies are warranted to establish their robustness and clarify whether therapeutic modulation can improve outcomes.

## Supplementary Information

Additional file 1.

## Data Availability

The data sets used and/or analyzed during the current study are available from the corresponding author on reasonable request.

## Abbreviations

AB: Admission blood gas value
ABG: Admission blood glucose
ABGL: Admission blood glucose–lactate ratio
ABL: Admission blood lactate
ARDS: Acute respiratory distress syndrome
AUC: Area under the curve
BGA: Blood gas analysis
BMI: Body mass index
CI: Confidence interval
CKD: Chronic kidney disease
COPD: Chronic obstructive pulmonary disease
CV: Coefficient of variation
CVG: Coefficient of variation for glucose
CVGL: Coefficient of variation for glucose–lactate ratio
CVL: Coefficient of variation for lactate
FPR: False positive rate
GCS: Glasgow coma scale
GM: German modification
ICD: International Classification of Diseases
ICU: Intensive care unit
ip-LC: Interpolated lactate clearance
IQR: Interquartile range
ISS: Injury severity score
LOS: Length of stay
MB: Mean blood gas value index
MBG: Mean blood glucose
MBGL: Mean blood glucose–lactate ratio
MBL: Mean blood lactate
non-c-SOFA: Sequential organ failure assessment score exclusive cranial injury (within 24 h after ICU admission)
OR: Odds ratio
PSM: Propensity score matching
raw-LC: Raw value lactate clearance
RCS: Restricted cubic splines
ROC: Receiver operating characteristic
SAPS II: Simplified acute physiology score II
SD: Standard deviation
SOFA: Sequential organ failure assessment score
STROBE: Strengthening the Reporting of Observational Studies in Epidemiology
TBI: Traumatic brain injury
TPR: True positive rate
TUDR: Time-unified dysglycemic rate
TWA: Time-weighted average index
TWAG: Time-weighted average glucose
TWAGL: Time-weighted average glucose–lactate ratio
TWAL: Time-weighted average lactate
VIF: Variance inflation factor
