# Supplementary material for: Time-weighted lactate and glucose–lactate ratio outperform static values in ICU mortality prediction after traumatic brain injury: a retrospective cohort study
Source: J Intensive Care. 2026 Feb 7;14:26. doi: 10.1186/s40560-026-00864-9 (PMC12977792; doi:10.1186/s40560-026-00864-9)
Supplement: Supplementary file 1 — Additional file 1. [file 40560_2026_864_MOESM1_ESM.pdf]

Supplementary material

Time-weighted lactate and glucose-lactate ratio outperform static values in ICU mortality prediction after traumatic brain injury: a retrospective cohort study

Matthias Manfred Deininger[1#*]; Magdalena Ralser[1#]; Nico Haehn[1]; Marius Huehn[1]; Dmitrij Ziles[1]; Gernot Marx[1]; Catharina Conzen-Dilger[2]; Anke Hoellig[2]; Thomas Breuer[1]

Affiliations:

[1] Department of Intensive Care Medicine, Faculty of Medicine, RWTH Aachen University, Aachen, Germany

[2] Department of Neurosurgery, Faculty of Medicine, RWTH Aachen University, Aachen, Germany

[#] Both authors contributed equally

* Corresponding author: mdeininger@ukaachen.de

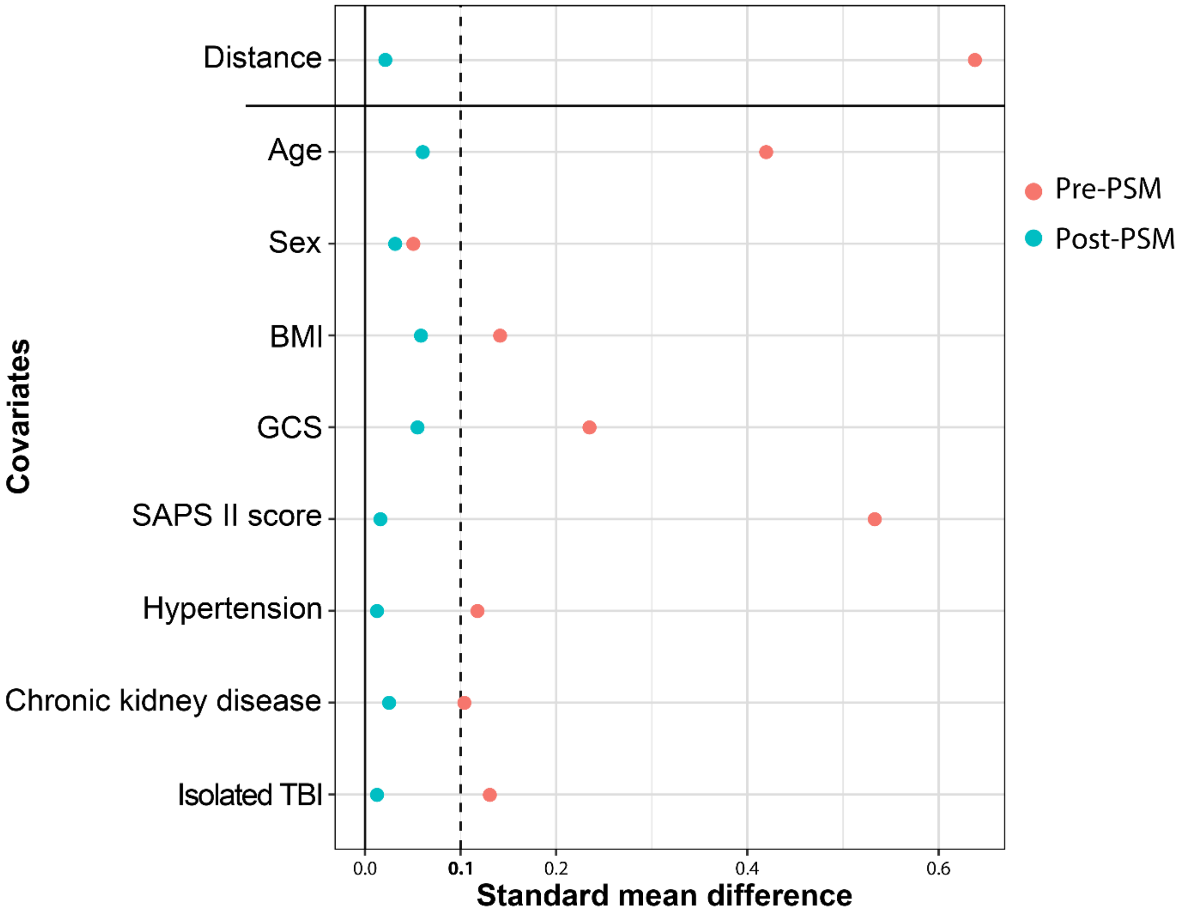

Figure S1: Love plot for propensity score matching

Comparison of the standard mean differences before (pre-PSM, red color) and after (post-PSM, green color) propensity score matching (PSM) is shown. Standard mean difference is shown on the x-axis, the covariates and the distance are displayed on the y-axis. The target level of 0.1 is illustrated by a vertical dashed line.

BMI: Body mass index; GCS: Glasgow coma scale; ICU: Intensive care unit; SAPS II: Simplified acute physiology score II; TBI: Traumatic brain injury

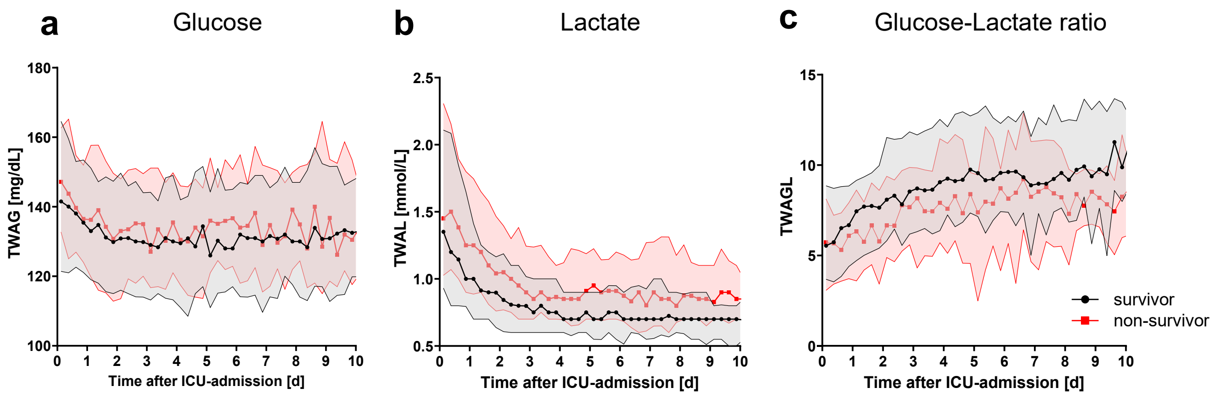

Figure S2: Trend of glucose and lactate over ICU stay (raw data)

The figure shows the trend over time of a) glucose (TWAG), b) lactate (TWAL), and c) the glucose-lactate ratio (TWAGL) over the first ten ICU days, stratified by survival status. The time is shown in days on the x-axis and the corresponding measured value on the y-axis. The median 6h-time weighted values are shown here as connected dots. Shaded ribbons represent the interquartile range.

ICU: Intensive care unit; mg/dL: Milligram per deciliter; mmol/L: Millimole per Liter; TWAG: Time-weighted average glucose; TWAGL: Time-weighted average glucose-lactate ratio; TWAL: Time-weighted average lactate

Table S1: Additional lactate clearance indices

	Survivor *n=150*	*Non-survivor* *n=79*	*p-value*
**LACTATE**			
6h-raw-LC [%]	5.1 (-16.8-32.6)	11.1 (-18.2-33.3)	0.722
6h-ip-LC [%]	12.1 (-22.9-35.2)	8.2 (-21.7-31.5)	0.721
24h-raw-LC [%]	25.0 (-14.6-54.7)	16.7 (-20.0-44.0)	0.376
24h-ip-LC [%]	27.7 (-11.0-55.1)	18.4 (-19.4-40.1)	0.284
72h-raw-LC [%]	50.0 (20.0-67.7)	38.5 (0.0-68.2)	0.202

Data are presented as median (IQR). Negative lactate clearance (LC) values correspond to a percentage increase, positive values to a decrease in lactate over time.

ip-LC: Interpolated lactate clearance; raw-LC: Raw value lactate clearance

Figure S3: Pairwise Spearman correlation matrix

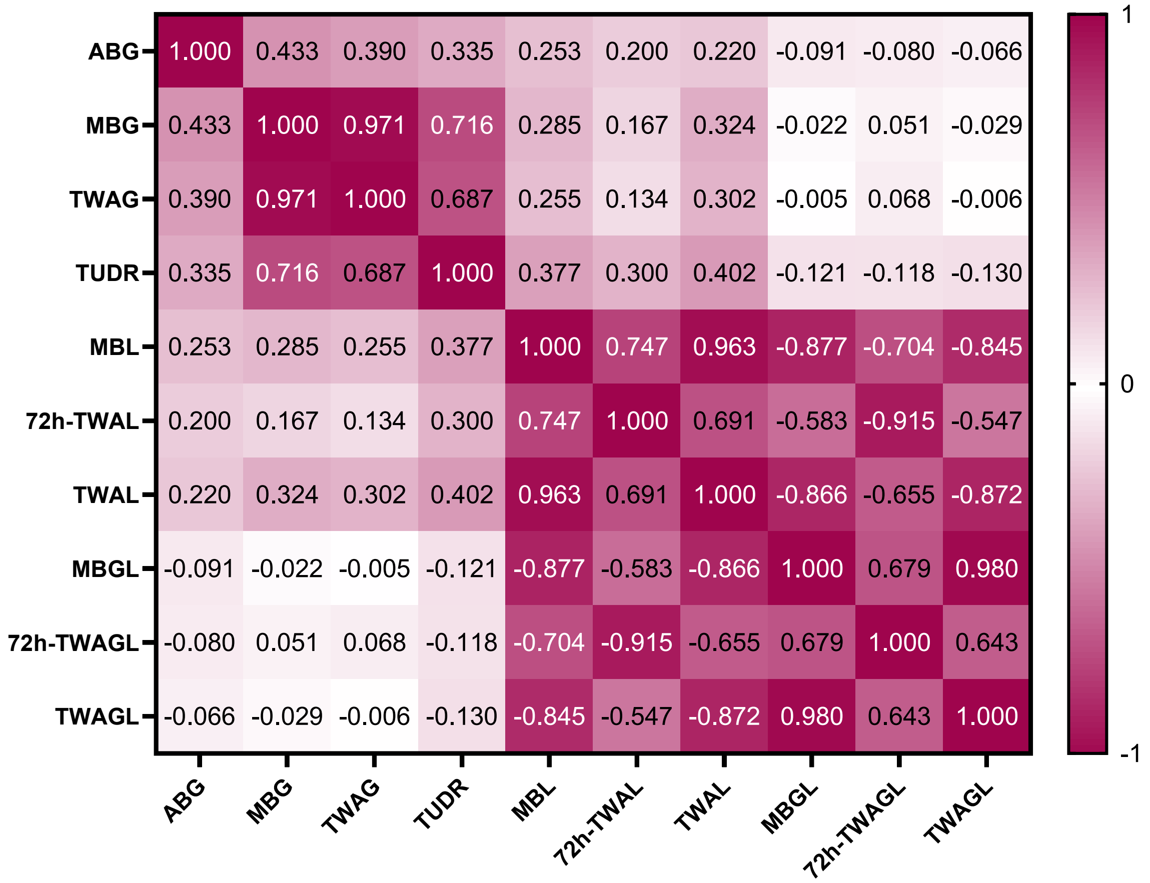

Displayed is the pairwise Spearman correlation as a heat map. Higher color intensity is associated with a higher Spearman correlation coefficient. Effect size |≥0.500| was defined as high collinearity in the two-sided Pearson correlation.

ABG: Admission blood glucose; MBG: Mean blood glucose; MBGL: Mean blood glucose-lactate ratio; MBL: Mean blood lactate; TUDR: Time-unified dysglycemic rate; TWAG: Time-weighted average glucose; TWAGL: Time-weighted average glucose-lactate ratio; TWAL: Time-weighted average lactate

Table S2: Variance inflation factor summary as collinearity check for multivariable models

Variables	Glucose & Lactate		Glucose-Lactate-ratio	
	First 72h	Total ICU duration	First 72h	Total ICU duration
Sex (male)	1.044	1.037	1.042	1.023
SAPS II Score	1.041	1.057	1.018	1.023
ABG	1.112	1.268		
TWAG		1.275		
TWAL		1.031		
72h-TWAL	1.104			
TWAGL				1.009
72h-TWAGL			1.024	

Variance inflation factor (VIF) was assessed separately for all multivariable models reported in the manuscript.

ABG: Admission blood glucose; SAPS II: Simplified acute physiology score II; TWAG: Time-weighted average glucose; TWAGL: Time-weighted average glucose-lactate ratio; TWAL: Time-weighted average lactate

Table S3: Conditional logistic regression (clogit) stratified by match set

Variables	Glucose & Lactate						Glucose-Lactate-ratio					
	First 72h			Total ICU duration			First 72h			Total ICU duration		
	**OR [95%-CI]**	**p-value**	**z-score**	**OR [95%-CI]**	**p-value**	**z-score**	**OR [95%-CI]**	**p-value**	**z-score**	**OR [95%-CI]**	**p-value**	**z-score**
SAPS II Score	0.983 [0.939-1.029]	0.470	-0.723	0.974 [0.923-1.029]	0.347	-0.941	0.992 [0.949-1.038]	0.735	-0.339	0.989 [0.944-1.036]	0.645	-0.460
ABG	1.007 [0.999-1.014]	0.088	1.704	1.006 [0.997-1.015]	0.185	1.325						
TWAG				1.016 [0.994-1.038]	0.158	1.413						
TWAL				**14.033 [4.432-44.431]**	**<0.001**	**4.492**						
72h-TWAL	**2.213 [1.291-3.792]**	**0.004**	**2.891**									
TWAGL										**0.760 [0.664-0.870]**	**<0.001**	**-3.995**
72h-TWAGL							**0.836 [0.746-0.937]**	**0.002**	**-3.087**			

Multivariable logistic regression models were adjusted for baseline covariates. Odds ratios (OR) were reported separately for the two analyzed time horizons (first 72 h and full ICU stay). Only non-redundant indices were used to minimize collinearity. Data with significant p-values are shown in bold.

ABG: Admission blood glucose; SAPS II: Simplified acute physiology score II; TWAG: Time-weighted average glucose; TWAGL: Time-weighted average glucose-lactate ratio; TWAL: Time-weighted average lactate

Sex not estimable/insufficient within-set variation therefore not shown.

Table S4: Conventional logistic regression (Wald)

Variables	Glucose & Lactate						Glucose-Lactate-ratio					
	First 72h			Total ICU duration			First 72h			Total ICU duration		
	**OR [95%-CI]**	**p-value**	**z-score**	**OR [95%-CI]**	**p-value**	**z-score**	**OR [95%-CI]**	**p-value**	**z-score**	**OR [95%-CI]**	**p-value**	**z-score**
Sex (male)	0.896 [0.469-1.712]	0.739	-0.333	1.003 [0.506-1.990]	0.993	0.009	0.944 [0.496-1.797]	0.861	-0.175	0.910 [0.477-1.738]	0.776	-0.285
SAPS II Score	1.000 [0.969-1.032]	0.998	0.002	0.991 [0.957-1.026]	0.613	-0.506	1.004 [0.974-1.036]	0.791	0.265	1.000 [0.969-1.033]	0.981	0.023
ABG	1.005 [0.998-1.013]	0.145	1.457	1.003 [0.994-1.011]	0.528	0.632						
TWAG				1.015 [0.995-1.036]	0.145	1.459						
TWAL				**14.701 [4.718-45.812]**	**<0.001**	**4.635**						
72h-TWAL	**2.127 [1.259-3.596]**	**0.005**	**2.819**									
TWAGL										**0.754 [0.661-0.859]**	**<0.001**	**-4.221**
72h-TWAGL							**0.835 [0.745-0.935]**	**0.002**	**-3.119**			

Multivariable logistic regression models were adjusted for baseline covariates. Odds ratios (OR) were reported separately for the two analyzed time horizons (first 72 h and full ICU stay). Only non-redundant indices were used to minimize collinearity. Data with significant p-values are shown in bold.

ABG: Admission blood glucose; SAPS II: Simplified acute physiology score II; TWAG: Time-weighted average glucose; TWAGL: Time-weighted average glucose-lactate ratio; TWAL: Time-weighted average lactate

Table S5: Post-baseline-adjusted logistic regression including septic shock and acute kidney injury for full ICU stay

Variables	CR2			Clogit*			Wald		
	**OR [95%-CI]**	**p-value**	**z-score**	**OR [95%-CI]**	**p-value**	**z-score**	**OR [95%-CI]**	**p-value**	**z-score**
Sex (male)	1.009 [0.448-2.273]	0.982	0.023				1.009 [0.505-2.018]	0.979	0.026
Septic shock	1.152 [0.448-2.964]	0.769	0.294	1.104 [0.372-3.274]	0.859	0.178	1.152 [0.456-2.914]	0.765	0.299
Acute kidney injury	**3.153 [1.221-8.142]**	**0.018**	**2.373**	**3.096 [1.042-9.200]**	**0.042**	**2.034**	**3.153 [1.160-8.567]**	**0.024**	**2.252**
SAPS II Score	0.988 [0.962-1.014]	0.357	-0.922	0.942 [0.884-1.004]	0.066	-1.841	0.988 [0.953-1.024]	0.497	-0.679
ABG	1.004 [0.994-1.014]	0.465	0.731	1.006 [0.997-1.016]	0.170	1.371	1.004 [0.995-1.013]	0.390	0.860
TWAG	1.016 [0.993-1.041]	0.181	1.338	1.016 [0.994-1.038]	0.154	1.426	1.016 [0.995-1.038]	0.128	1.523
TWAL	**12.204 [4.500-33.097]**	**<0.001**	**4.915**	**13.433 [4.082-44.210]**	**<0.001**	**4.274**	**12.204 [3.858-38.603]**	**<0.001**	**4.258**

Multivariable logistic regression models were adjusted for baseline covariates. Data with significant p-values are shown in bold.

ABG: Admission blood glucose; SAPS II: Simplified acute physiology score II; TWAG: Time-weighted average glucose; TWAL: Time-weighted average lactate, CR2: Cluster-robust variance at the match-set level logistic regression model, Clogit: Conditional logistic regression stratified by match set, Wald: Conventional logistic regression based on Wald test

* Sex not estimable/insufficient within-set variation therefore not shown

Table S6: Logistic regression (Cluster-robust variance at the match-set level) – including isolated-TBI as covariate

Variables	Glucose & Lactate						Glucose-Lactate-ratio					
	First 72h			Total ICU duration			First 72h			Total ICU duration		
	OR [95%-CI]	p-value	z-score	OR [95%-CI]	p-value	z-score	OR [95%-CI]	p-value	z-score	OR [95%-CI]	p-value	z-score
Sex (male)	0.885 [0.420-1.867]	0.749	-0.320	0.984 [0.434-2.229]	0.969	-0.039	0.933 [0.444-1.957]	0.854	-0.184	0.893 [0.422-1.889]	0.768	-0.295
Isolated-TBI	1.250 [0.689-2.267]	0.463	0.734	1.413 [0.787-2.537]	0.247	1.157	1.282 [0.722-2.276]	0.396	0.849	1.241 [0.723-2.128]	0.433	0.784
SAPS II Score	0.999 [0.975-1.023]	0.943	-0.072	0.989 [0.961-1.018]	0.444	-0.765	1.003 [0.980-1.027]	0.780	0.279	0.999 [0.975-1.024]	0.961	-0.049
ABG	1.005 [0.998-1.013]	0.173	1.361	1.003 [0.993-1.013]	0.583	0.549						
TWAG				1.015 [0.992-1.039]	0.193	1.303						
TWAL				**15.780 [5.785-43.045]**	**<0.001**	**5.388**						
72h-TWAL	**2.240 [1.351-3.713]**	**0.002**	**3.125**									
TWAGL										**0.748 [0.655-0.853]**	**<0.001**	**-4.328**
72h-TWAGL							**0.823 [0.726-0.933]**	**0.002**	**-3.043**			

Multivariable logistic regression models were adjusted for baseline covariates. Odds ratios (OR) were reported separately for the two analyzed time horizons (first 72 h and full ICU stay). Only non-redundant indices were used to minimize collinearity. Data with significant p-values are shown in bold.

ABG: Admission blood glucose; SAPS II: Simplified acute physiology score II; TBI: Traumatic brain injury; TWAG: Time-weighted average glucose; TWAGL: Time-weighted average glucose-lactate ratio; TWAL: Time-weighted average lactate

Table S7: Logistic regression (Cluster-robust variance at the match-set level) – including isolated-TBI and non-c-SOFA as covariates

Variables	Glucose & Lactate						Glucose-Lactate-ratio					
	First 72h			Total ICU duration			First 72h			Total ICU duration		
	**OR [95%-CI]**	**p-value**	**z-score**	**OR [95%-CI]**	**p-value**	**z-score**	**OR [95%-CI]**	**p-value**	**z-score**	**OR [95%-CI]**	**p-value**	**z-score**
Sex (male)	0.875 [0.404-1.897]	0.735	-0.339	0.937 [0.400-2.191]	0.880	-0.151	0.937 [0.434-2.024]	0.869	-0.166	0.884 [0.405-1.932]	0.757	-0.309
Isolated-TBI	1.229 [0.677-2.233]	0.498	0.678	1.329 [0.737-2.394]	0.344	0.946	1.278 [0.719-2.273]	0.403	0.837	1.221 [0.715-2.086]	0.465	0.731
Non-c-SOFA	0.979 [0.861-1.113]	0.745	-0.326	0.957 [0.834-1.099]	0.537	-0.617	0.990 [0.875-1.120]	0.874	-0.158	0.983 [0.866-1.114]	0.783	-0.275
ABG	1.005 [0.998-1.013]	0.168	1.377	1.003 [0.993-1.013]	0.570	0.568						
TWAG				1.014 [0.991-1.038]	0.223	1.220						
TWAL				**16.476 [5.982-45.384]**	**<0.001**	**5.420**						
72h-TWAL	**2.296 [1.390-3.792]**	**0.001**	**3.247**									
TWAGL										**0.744 [0.650-0.852]**	**<0.001**	**-4.277**
72h-TWAGL							**0.821 [0.724-0.930]**	**0.002**	**-3.091**			

Multivariable logistic regression models were adjusted for baseline covariates. Odds ratios (OR) were reported separately for the two analyzed time horizons (first 72 h and full ICU stay). Only non-redundant indices were used to minimize collinearity. Data with significant p-values are shown in bold.

ABG: Admission blood glucose; non-c-SOFA: Sequential organ failure assessment score exclusive cranial injury (within 24h after ICU admission); SAPS II: Simplified acute physiology score II; TBI: Traumatic brain injury; TWAG: Time-weighted average glucose; TWAGL: Time-weighted average glucose-lactate ratio; TWAL: Time-weighted average lactate

Table S8: Logistic regression (Cluster-robust variance at the match-set level) – including isolated-TBI, non-c-SOFA and for non-isolated TBI patients ISS as covariates

Variables	Glucose & Lactate						Glucose-Lactate-ratio					
	First 72h			Total ICU duration			First 72h			Total ICU duration		
	**OR [95%-CI]**	**p-value**	**z-score**	**OR [95%-CI]**	**p-value**	**z-score**	**OR [95%-CI]**	**p-value**	**z-score**	**OR [95%-CI]**	**p-value**	**z-score**
Sex (male)	0.922 [0.422-2.018]	0.840	-0.202	0.965 [0.412-2.259]	0.935	-0.081	0.988 [0.453-2.153]	0.976	-0.030	0.922 [0.421-2.022]	0.840	-0.202
Isolated-TBI	1.222 [0.662-2.257]	0.521	0.641	1.314 [0.725-2.384]	0.368	0.900	1.279 [0.708-2.311]	0.416	0.814	1.222 [0.705-2.117]	0.476	0.713
Non-c-SOFA	0.982 [0.862-1.118]	0.785	-0.273	0.958 [0.834-1.102]	0.550	-0.597	0.993 [0.876-1.125]	0.910	-0.113	0.983 [0.865-1.118]	0.799	-0.255
ISS	1.491 [0.952-2.335]	0.081	1.743	1.283 [0.725-2.270]	0.393	0.854	1.454 [0.942-2.243]	0.091	1.691	1.359 [0.888-2.079]	0.158	1.412
ABG	1.006 [0.998-1.014]	0.134	1.500	1.003 [0.993-1.013]	0.565	0.576						
TWAG				1.015 [0.992-1.039]	0.204	1.269						
TWAL				**15.539 [5.563-43.406]**	**<0.001**	**5.234**						
72h-TWAL	**2.077 [1.217-3.546]**	**0.007**	**2.681**									
TWAGL										**0.756 [0.659-0.868]**	**<0.001**	**-3.986**
72h-TWAGL							**0.835 [0.736-0.949]**	**0.006**	**-2.773**			

Multivariable logistic regression models were adjusted for baseline covariates. Odds ratios (OR) were reported separately for the two analyzed time horizons (first 72 h and full ICU stay). Only non-redundant indices were used to minimize collinearity. Data with significant p-values are shown in bold.

ABG: Admission blood glucose; ISS: Injury severity score; non-c-SOFA: Sequential organ failure assessment score exclusive cranial injury (within 24h after ICU admission); SAPS II: Simplified acute physiology score II; TBI: Traumatic brain injury; TWAG: Time-weighted average glucose; TWAGL: Time-weighted average glucose-lactate ratio; TWAL: Time-weighted average lactate
